# Differential Anti-Proliferative and Anti-Migratory Activities of Ursolic Acid, 3-*O*-Acetylursolic Acid and Their Combination Treatments with Quercetin on Melanoma Cells

**DOI:** 10.3390/biom10060894

**Published:** 2020-06-11

**Authors:** Aljawharah AlQathama, Luying Shao, Ammar Bader, Proma Khondkar, Simon Gibbons, Jose M Prieto

**Affiliations:** 1 School of Pharmacy, University College London, London WC1N 1AX, UK; luying.shao.12@ucl.ac.uk (L.S.); Proma.khondkar@ncclondon.ac.uk (P.K.); S.Gibbons@uea.ac.uk (S.G.); 2 Department of Pharmacognosy, Pharmacy College, Umm Al-Qura University, Makkah 21955, Saudi Arabia; ambader@uqu.edu.sa; 3 School of Pharmacy, University East Anglia, Norwich NR4 7TJ, UK; 4 Centre for Natural Products Discovery, School of Pharmacy and Biomolecular Sciences, Liverpool John Moores University, Liverpool L3 3AF, UK

**Keywords:** pentacyclic triterpenes, apoptosis, melanoma, migration, cell cycle, synergism

## Abstract

We evaluate how 3-acetylation modulates the in vitro activity of ursolic acid in melanoma cells alone or in combination treatments with quercetin. Anti-proliferative studies on A375 cells and adult human dermal fibroblasts included analyses on cell cycle distribution, caspase activity, phosphatidylserine translocation, cell morphology and Bax/Bcl-2 protein expression. Then, 2D and 3D migration of B16F10 cells were studied using scratch and Transwell assays, respectively. Ursolic acid and 3-*O*-acetylursolic acid have shown similar GI_50_ on A375 cells (26 µM vs. 32 µM, respectively) significantly increased both early and late apoptotic populations, activated caspases 3/7 (48–72 h), and enhanced Bax whilst attenuating Bcl-2 expression. Ursolic acid caused elevation of the sub-G1 population whilst its 3-acetyl derivative arrested cell cycle at S phase and induced strong morphological changes. Combination treatments showed that ursolic acid and quercetin act synergistically in migration assays but not against cell proliferation. In summary, 3-*O*-acetylursolic acid maintains the potency and overall apoptotic mechanism of the parent molecule with a more aggressive influence on the morphology of A375 melanoma cells but the 3-acetylation suppresses its anti-migratory properties. We also found that ursolic acid can act in synergy with quercetin to reduce cell migration.

## 1. Introduction

Pentacyclic terpenes such as betulinic acid, lupeol, ursolic acid, and oleanolic acids have been described as highly specific cytotoxic drugs against several cancer cells [1,2,3,4,5].

The effects of ursolic acid on melanoma cells has been gaining interest for its cytotoxic with a favourable selectivity index [6,7,8]. Moreover, it also suppresses the migration of malignant melanoma cells [9]. Clinical tests suggesting the possibility of practical use of ursolic acid have already been conducted [10].

**Figure 1:**
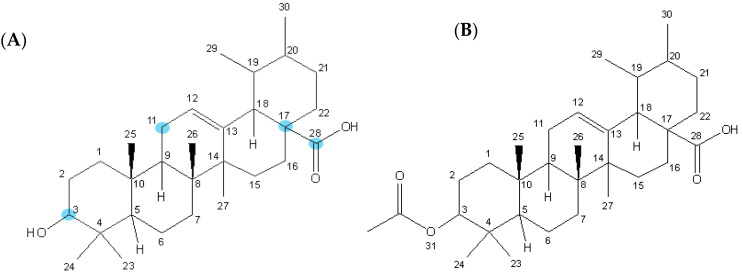
Chemical structure of ursolic acid (**A**) and 3-*O*-acetylursolic acid (**B**).

Derivatization of terpenoids might improve its cytotoxicity. In the case of ursolic acid, the anti-proliferative effect was improved when a hydrogen acceptor group was introduced to positions 3, 11, 17 and 28 like acetyl or aminoalkyl groups, with β-oriented hydrogen-bond forming groups at C-3 exhibiting more potent cytotoxicity than their α-counterparts (Figure 1) [11,12,13,14]. However, modification at position 28, namely amidation and esterification carboxylic acid, led to a decrease of its selectivity index [15]. 

The simplicity of the 3-acetyl derivative, commonly referred to as acetylursolic acid or 3-*O*-acetylursolic acid, makes it a good candidate for further mechanistic studies and has not yet been studied for its anti-migratory activities. Previous works studied the semisynthetic product. It seems that 3-*O*-acetylursolic acid is scarce in nature as it has been seldom isolated. Some of its reported sources are the aerial roots of *Ficus microcarpa* (Moraceae)*,* the leaves of *Carissa spinarum* L. (Apocynaceae) [16], and the aerial parts of *Strumpfia maritima* Jacq. (Rubiaceae) [17]. 

Another way to increase the apoptotic and anti-migratory effects of ursolic acid is by combination with a synergic drug. Quercetin is a secondary metabolite that is known for its anti-migratory activity and shown synergistic activity with sulforaphane [18]. Many studies suggest that ursolic acid and quercetin not only can be used as cancer chemosensitizers to standard chemotherapeutic drugs but also as chemoprotective and radioprotective thus protecting normal cells [19,20]. Their combination would, therefore, provide notable advantages in anticancer treatment. 

Therefore, we here aim to contribute to the current knowledge of the effects of pentacyclic triterpenes acids on melanoma cells by studying the antimigratory activity and pro-apoptotic mechanisms of its 3-acetyl derivative as well as the effects of combinatorial treatments of ursolic acid and quercetin on cell proliferation and 2D/3D migration.

## 2. Materials and Methods 

### 2.1. Materials

Ursolic acid and its acetate were isolated as previously described [16]. Other chemicals were purchased from Sigma-Aldrich (St. Louis, MO, USA): ursolic acid (≥90%) and quercetin (≥90%), Sulforhodamine B, trichloroacetic acid, Trizma base, propidium iodide, Ribonuclease A, formaldehyde, and crystal violet. Glacial acetic acid, ethanol, and methanol were obtained from Fisher (Leicestershire, UK). Dulbecco’s modified eagle media (DMEM), minimum essential media (MEM), heat-inactivated fetal bovine serum (FBS), penicillin-streptomycin antibiotic, non-essential amino acids solution (NEAA), TrypLE Express (1×, trypsin, EDTA, phenol red), phosphate-buffered saline (PBS), ReadyProbes^®^ cell viability imaging kittrypan blue were purchased from Thermo Fisher Scientific (Waltham, MA, USA). Matrigel was purchased from BD Bioscience (San Jose, CA, USA), SDS-PAGE gel from Bio-Rad (Hercules, CA, USA), Caspase-Glo^®^ 3/7 from Promega, Annexin V-FITC kit from Miltenyi Biotec and Bax, Bcl-2 and β-actin proteins from Cell Signaling Technology (Danvers, MA, USA).

### 2.2. Cell Lines

A375 (human malignant melanoma) and B16-F10 (murine malignant melanoma) cell lines were purchased from American Type Culture Collection (Manassas, VA, USA) and HDf-a (primary adult human dermal fibroblasts) from Thermo Fisher Scientific (Waltham, MA, USA). A375 and HDf-a were used to study the cytotoxicity and selectivity of compounds and B16-F10 cell line was used in the scratch and Boyden chamber assays. A375 cells were maintained in DMEM and supplemented with 10% FBS and 1% penicillin-streptomycin antibiotic. HDf-a cells were grown in MEM supplemented with 10% FBS, 1% MEM-NEAA, and 1% antibiotic solution. The media used to maintain B16-F10 was MEM, supplemented with 10% FBS and 1% of the antibiotic solution. All cell lines were cultured in complete growth medium (10% FBS) and incubated in an incubator with humidified air 5% CO_2_ and atmosphere at 37 °C.

### 2.3. Sulforhodamine B (SRB) Assay

This assay was conducted as previously described [21], A375 and HDF-a cells were seeded in a 96-well microtiter plate at a density of 10,000 cells per well to allow the cells to attach to the plate. Then, cells were treated with different concentrations of isolated compounds with vehicle control (DMSO) which had been previously prepared in 10% culture medium. The cells were incubated in the incubator for 24, 48, and 72 h and periodically checked using an inverted microscope. Later, the cells were fixed with cold 40% trichloroacetic acid (TCA) solution, to achieve the final concentration of 10%. The plates were incubated at 4 °C for 1 h and then rinsed five times with water. The TCA-fixed cells were stained by adding Sulforhodamine B solution (0.4% SRB in 0.1% acetic acid) and left at room temperature for 1 h. Afterwards, the plates were quickly rinsed four times with 1% acetic acid and flicked to remove the unbound dye and then left to air-dry overnight. The bounded stain was solubilised by adding 10 mM Tris base buffer solution to each well. The optical density was measured at 510 nm by using a microtiter plate reader (Infinite^®^ M200, Tecan, Switzerland). The data was normalized to untreated wells, GI_50_ value was calculated as the concentration that results in 50% cell growth inhibition and graphs were drawn on OriginPro software. 

### 2.4. Cell Cycle Analysis

The cell distribution at different stages of the cell cycle was measured through cellular DNA analysis and performed using A375 cells according to the method of Li and colleagues [22]. The cells were seeded at a density of 500,000 cells in serum-free medium in a 6-well plate and left to attach in the incubator at 37 °C overnight. Compounds and DMSO in 10% growth media were added after removing the old media and incubated for 48 h. Afterwards, cells were washed with PBS and detached by adding TrypLE and combined with floated cells. Then, the cell suspension was centrifuged and washed twice with PBS. Later, cells were fixed by cold 70% ethanol in PBS and incubated for 18 h at 4 °C. After removing the ethanol, the cells were washed and treated with 100 µg/mL of RNase for 30 min. In the dark, they were stained with propidium iodide (PI) solution, 50 µg/mL of PI dissolved in PBS, and incubated for 10 min. The sample was analysed by MACSQuant Analyzer and MACSQuantify™ software (Miltenyi Biotech, Germany). The assay was carried out in three independent experiments (mean ± SD, n = 3).

### 2.5. Apoptosis Induction Analysis by Flow Cytometry

Analysis of phosphatidylserine translocation was performed using Annexin V-FITC kit according to the manufacturer’s instructions (Miltenyi Biotec, Germany). Around 250,000 of A375 cells were seeded in a 12-well plate overnight. Later, 10% growth media containing compound and DMSO were added and incubated for 12 and 24 h at 37 °C. After the end of the incubation period, cells were detached using TrypLE and washed with PBS and next with the binding buffer. Then, the cell pellet was resuspended in binding buffer containing Annexin V-FITC and incubated for 15 min in the dark at room temperature. Later, cells were incubated with binding buffer containing 1 µg/mL of propidium iodide (PI) solution immediately before analysis by flow cytometry. The MACSQuant^®^ Analyzer was used for data acquisition and the MACSQuantify software for data analysis and data was applied from three independent experiments across different cell passages (mean ± SD, n = 3).

### 2.6. Caspase-3/7 Activity Assay

The apoptosis induced by compounds was determined by measuring the activity of caspases-3/7 using Caspase-Glo^®^ 3/ according to the manufacturer’s protocol (Promega, Madison, WI, USA). 10,000 A375 cells were seeded in a 96-well plate at and treated with compounds or DMSO for different time points (24, 48 and 72 h). Afterwards, 100 μL of culture media of each well was transferred to a 96 white multi-well plate and 100 μL of Caspase-Glo^®^ 3/7 reagent was added and incubated for 1 h at room temperature. When the incubation period was completed, the luminescence of each sample representing the enzymatic activity of caspase was measured using a plate-reading luminometer (Tecan, Switzerland). The assay was performed in three independent experiments and each sample was performed in triplicate (mean ± SD, n = 9).

### 2.7. Phase-Contrast and Fluorescence Microscopy

Morphological assessment of A375 cells was performed to detect the cellular changes induced by the tested compounds according to the previously mentioned method, with slight modifications [23]. A375 cells were seeded at a density of 10,000 cells in a sterile chamber slide system (Lab-Tek™ II chamber slide™ system, Nunc™, Rochester, NY, USA) with or without compounds/DMSO for 48 h. Treated cells with typical morphological changes of apoptosis were imaged using a phase-contrast inverted microscope (EVOS cell imaging system, Thermo Fisher Scientific, Waltham, MA, USA). 

A375 cells were treated in the same condition and examined with a fluorescence microscope using the ReadyProbes^®^ cell viability imaging kit according to the manufacturer’s recommendations (Thermo Fisher Scientific, Waltham, MA, USA). The dyes were added and incubated at 37 °C for 15 min and the wells were visualised using the EVOS cell imaging system. The results were obtained by capturing images for six random fields for each sample from three different experiments.

### 2.8. Western Blot Analysis

Changes of protein expression were investigated using Western blot application solution kit according to the manufacturer’s recommendation (Cell Signalling Technology, Danvers, MA, USA). After seeding A375 cells and treating them with compounds and DMSO for 48 h, cells were lysed, sonicated and centrifuged, the supernatant was used to quantify the protein concentration using a bicinchoninic acid (BCA) kit (Cell Signalling Technology, Danvers, MA, USA) as instructed by the manufacturer. The protein (50 µg) was suspended in SDS sample buffer and loaded to SDS-PAGE gel along with a prestained protein marker (11–190 kDa), in which electrophoresis was carried out at 110 V for 95 min (PowerPac™ Basic power Bio-Rad, Hercules, CA, USA). The gel was transferred into a nitrocellulose membrane in the same electrophoresis tank at 110 V for 95 min. After removing the gel, the membrane was blocked in blocking buffer for 1 h at room temperature. Then, the membrane was washed with TBST and probed with Bax, Bcl-2, and β-actin proteins, the latter was used as a standard for the amount of the loaded protein (50 µg) in each lane. Later, the bound primary antibody complex was stained using horseradish peroxidase-conjugated secondary antibodies. Protein bands were detected with enhanced chemiluminescence reagents and visualised by GeneGnome for chemiluminescence imaging (Syngene Bio Imaging, Frederick, MD, USA) using GENESys (version 1.3.3.0). Densitometric evaluation of change in expression levels of Bax/Bcl-2 proteins was carried out using GelQuantNET (version 1.8.2). Data were expressed as the ratio of Bax and Bcl-2 that was normalised to β-actin and the experiment was performed across three different passages (mean ± SD, n = 3).

### 2.9. Scratch Assay for Anti-Migratory Activity

This assay was carried out as described previously with slight modifications [24] using the B16-F10 cell line as it is a highly aggressive cell line to close the gap within a limited time. Around 250,000 cells were plated on a 6-well plate to produce a nearly confluent cell monolayer and later a scratch was made using a sterile 200 µL plastic pipette tip in the monolayer and wells were washed with the media to remove the detached cells. Free serum medium containing DMSO, the compounds and combinations were added and incubated for 24 h. Later, cells were fixed with 3.7% formaldehyde and representative images for each well were taken with EVOS cell imaging system. The images were analysed using TScratch software (ETH Zurich, Switzerland) and the potential synergistic effect of the combined drugs was analysed using CalcuSyn software (Biosoft, Cambridge, UK). Data were expressed as the migration rate of each sample relative to the control taken from four images over three independent experiments.

### 2.10. Transwell^©^ Assay for Anti-Invasive Activity

Cell invasion was also measured using a Boyden chamber system as described previously [25]. Polycarbonate filters with an 8-µm pore size (6.5 mm, Corning, NY, USA) pre-coated with Matrigel were placed to the top of Transwell filters in a 24-well plate. B16-F10 cells were seeded in serum-free medium onto the pre-coated filters and the lower chambers were filled with the same medium containing several concentrations of the drugs and incubated for 24 h. Later, the remaining cells were gently removed using a cotton swab and the inserts were washed and fixed with cold methanol. Then, the migrated cells were stained with 0.5% crystal violet for 30 min, then inserts were washed with PBS. The filter was cut out and mounted on a microscopic slide with a coverslip using VALAP (vaseline, lanolin, and paraffin) in which several images were taken using a light microscope (Nikon Microphot-FXA, Nikon, Tokyo, Japan) using a Nikon digital camera (Nikon, Kingston upon Thames, UK). The number of the cells that migrated to the lower side of the filter was determined using Infinity Capture software (version 6.2) and presented as a percentage of invaded cells/field compared to the control (mean ± SD) from three independent experiments.

### 2.11. Statistical Analysis

Results are expressed as mean ± standard deviation (SD) from at least three independent experiments. All data were analysed using unpaired two-tailed Student’s *t*-test with a *p*-value of <0.05 considered as significant to find the statistical significance between treated groups and controls using InStat v.3 (GraphPad, San Diego, CA, USA). Calcusyn software (Biosoft, Cambridge, UK) was used for the calculation of synergies/antagonisms by the Chou-Talalay method [26].

## 3. Results

### 3.1. Effects on A375 Human Melanoma Cells Proliferation and Selectivity Towards Adult Human Dermal Fibroblasts

**Figure 2:**
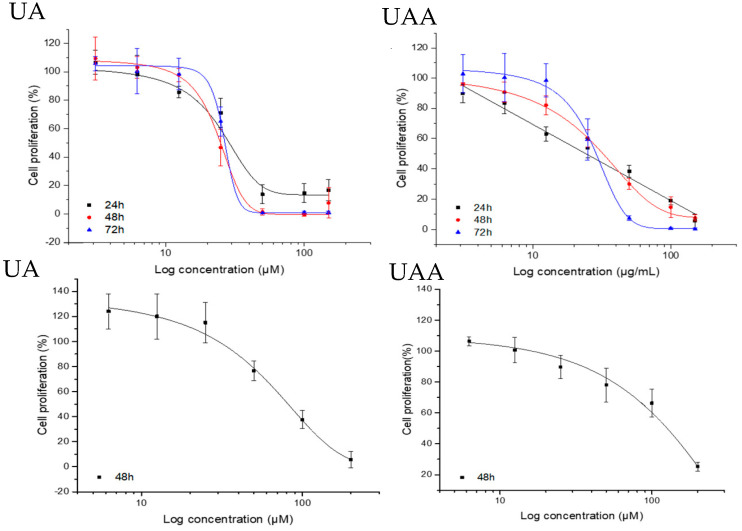
Anti-proliferative effect of 3-*O*-acetylursolic acid and ursolic acid on human melanoma A375 cells (upper row at 24, 48 and 72 h) and adult Human Dermal Fibroblasts HDf-a cells (lower row at 48 h) as assessed by the Sulforhodamine B assay. Data are presented as the mean ± SD (n = 3).

The anti-proliferative effects were studied using the SRB assay on both A375 and HDf-a cell lines (Figure 2). 3-*O*-acetylursolic acid and ursolic acid showed a significant time and concentration-dependent suppression of cell proliferation at 32.4 ± 1.33 µM and 26.7 ± 3.61 µM, respectively. They are comparatively non-toxic to HDf-a a shown by their relatively high GI_50_ concentrations (126.5 ± 24 µM for 3-*O*-acetylursolic acid and 89.31 ± 9.50 µM for ursolic acid). The selectivity of 3-*O*-acetylursolic acid and ursolic acid towards HDf-a compared to A375 cells were 4- and 3-fold, respectively.

### 3.2. Effects on A375 Cell Cycle Distribution

**Figure 3:**
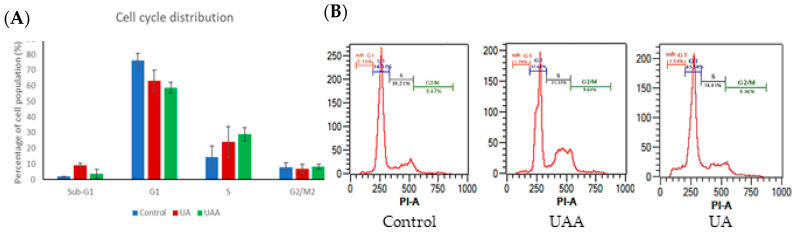
Cell-cycle analysis of human melanoma A375 cells exposed to GI_50_ concentrations of 3-*O*-acetylursolic acid (UAA) and ursolic acid (UA) at 48 h. (**A**) The percentage of cell population in each stage of cell cycle. (**B**) Cell cycle distribution (control and treated) was analysed by measuring the DNA content using flow cytometry. Data are shown as mean ± SD (n = 3).

Synchronised cells were assessed by flow cytometry after their exposure to GI_50_ doses of ursolic acid and its acetate for 48 h, to evaluate whether inhibition on cell proliferation is accompanied by any effect on cell cycle progression. Cell cycle distribution in 3-*O*-acetylursolic acid-treated cells showed a pronounced reduction of G1 population (58.70%, *p* = 0.0067) with a concomitant elevation of cell population at S stage (28.94%, *p* = 0.0373) compared to untreated cells (Figure 3A). Treatment of A375 cells with ursolic acid progressively increased the values of cell counts in sub-G1 phase (9.12%, *p* = 0.0008) which were considered to be apoptotic cells due to the decrease in their DNA content (Figure 3B).

### 3.3. Induction of Apoptosis According to Annexin V-FITC/Propidium Iodide (PI) Staining 

**Figure 4:**
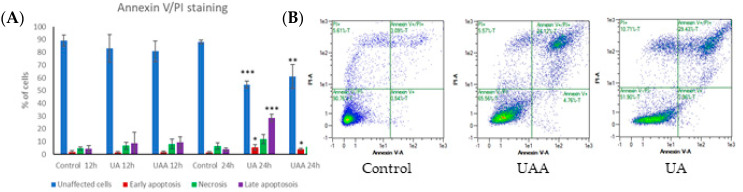
Flow cytometry analysis using Annexin V-FITC/propidium iodide (PI) double staining of human melanoma A375 cells incubated with 3-*O*-acetylursolic acid (UAA) and ursolic acid (UA) (GI_50,_ 12–24 h). (**A**) Quantification of unaffected, early/late apoptotic, and necrotic populations. (**B**) Apoptotic phases in control and treated cells. Data are presented as the mean ± SD (n = 3), (*) *p* < 0.05; (**) *p* < 0.01; (***) *p* < 0.001.

To investigate whether apoptosis was triggered following exposure to the compounds, phosphatidylserine translocation to the outer leaflet was examined by Annexin V-FITC and PI double staining, after treatment GI_50_ of both compounds for 12 and 24 h. After 12 h treatment, no significant effects were observed on the fluorescence intensity of Annexin V-FITC or PI for both compounds. Cellular exposure to 3-*O*-acetylursolic acid for 24 h led to an increase in the number of Annexin V-positive/ PI-negative cells (3.99%, *p* = 0.0118), which was concomitant with a decrease in the number of live cells (60.98%, *p* = 0.0077) (Figure 4A). Additionally, the percentage of double-positive population Annexin V-positive/PI-positive was significantly increased (29.06%, *p* = 0.0153). In the case of ursolic acid-treated cells, Figure 4B shows a significant Annexin V-positive/ PI-negative population (5.40%, *p* = 0.0420), which was associated with a reduction in the percentage of the normal population (54.65%, *p* < 0.0001). The magnitude of late apoptotic population (Annexin V-positive/ Pl-positive) was greatly increased (28.67%, *p* = 0.0002) compared to untreated cells. The number of necrotic (Annexin V-negative/ Pl-positive) cells was not significantly high and remained less than 10% for the entire period of the experiment. 

### 3.4. Changes in Cell Morphology Associated with the Treatment

**Figure 5:**
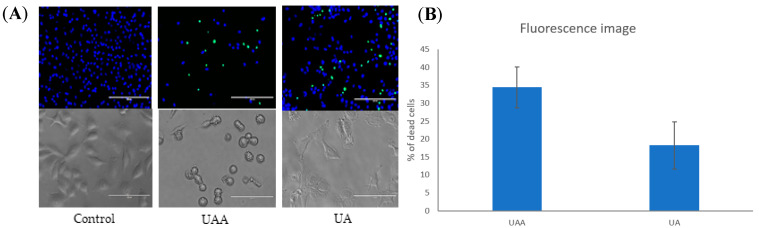
Microscopy analyses of human melanoma A375 cells after exposure to GI_50_ concentrations of 3-*O*-acetylursolic acid (UAA) and ursolic acid (UA) during 48 h. (**A**) Representative fluorescence images of stained cells with Hoechst 33342 and NucGreen^®^ Dead reagent and light contrast microscopy images (40×) of human melanoma A375 cells (control and treated). (**B**) Quantification of percentage of dead cells (green cells) from three independent experiments. Bar = 100 μm.

A qualitative evaluation of cell viability was conducted using the ReadyProbes^®^ cell viability imaging kit (blue/green) and phase contrast microscope to confirm that both compounds were cytotoxic to melanoma cells and to reflect the findings of the SRB assay. The percentage of green-stained cells after exposure to ursolic acid and its acetate for 48 h were elevated compared to the control, indicating that both compounds promoted cell death (Figure 5 upper row). Additionally, phase contrast images (Figure 5 lower row) show that untreated cells grew to near confluence and were able to spread regularly in the slide, whereas cells treated with 3-*O*-acetylursolic acid displayed several apoptotic features such as cell shrinkage and membrane blebbing over the incubation period. In the case of ursolic acid-treated cells, morphological changes were detected but to a lesser extent than 3-*O*-acetylursolic acid-treated cells, and this included a decrease in their size and a lack of cell-cell contact. Based on these findings, the dramatic changes in cell morphology provide evidence that the acetyl group may enhance the distinct morphological changes of apoptosis more in 3-*O*-acetylursolic acid-treated cells than in ursolic acid-treated cells.

### 3.5. Induction of Caspase-3/7 Activity

**Figure 6:**
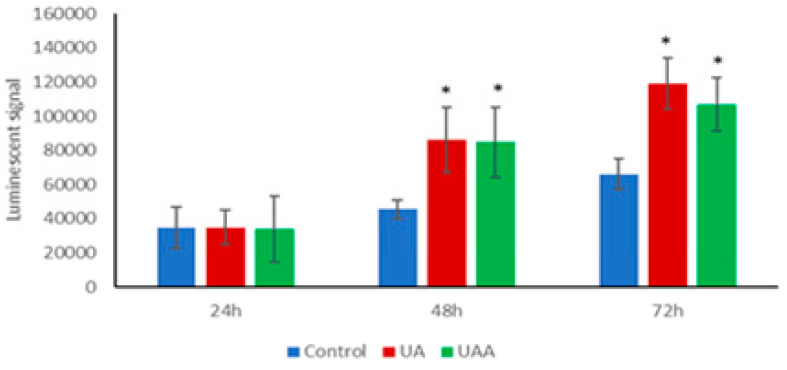
The caspase-3/7 activity of in human melanoma A375 cells treated with GI_50_ of 3-O-acetylursolic acid (UAA) and ursolic acid (UA) at different incubation periods (24, 48, and 72 h). Data are presented as the mean ± SD (n = 3), (*) *p* < 0.05.

As A375 cells underwent apoptosis after exposure to both compounds, the changes in caspase-3/7 were assessed where the cells were either treated with ursolic acid and its acetate, to evaluate whether the anti-proliferative activity associated with effects on caspase-mediated apoptosis pathway. Caspase-3/7 activity was induced upon cell exposure to the GI_50_ values with time-dependent enhancement in the treated cells. Both compounds promoted caspase-3/7 activity by 1.8-fold when compared to control cells (*p* < 0.05) at 48 h (Figure 6). 

### 3.6. Bax and Bcl-2 Expression in the Presence of Ursolic Acid and Its Acetate

**Figure 7:**
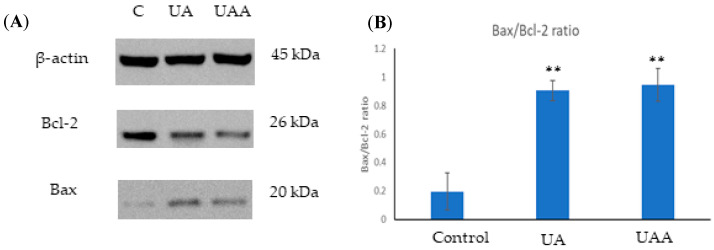
(**A**) Immunoblot images of the expression Bax and Bcl-2 in human melanoma A375 cells after 48 h treatment with GI_50_ of 3-*O*-acetylursolic acid (UAA) and ursolic acid (UA). (**B**) Densitometric evaluation of the expression levels of Bax/Bcl-2 proteins normalized to β-actin and changes in Bax/Bcl-2 ratio. Data are presented as the mean ± SD (n = 3), (**) *p* < 0.01.

Since Bax and Bcl-2 have a crucial role in apoptosis, Western blot analysis was performed to determine the effects of GI_50_ concentrations of 3-*O*-acetylursolic acid and ursolic acid on the expression level of both proteins in A375 cells. Following 48 h treatment, ursolic acid and its acetate caused an alteration in the expression of Bax and Bcl-2 on A375 cells, leading to a significant change in Bax/Bcl-2 ratio (*p* = 0.0017 and 0.0011, respectively). Bcl-2 was almost downregulated with simultaneous upregulation of Bax protein (Figure 7) in comparison to their respective controls.

**Figure 8:**
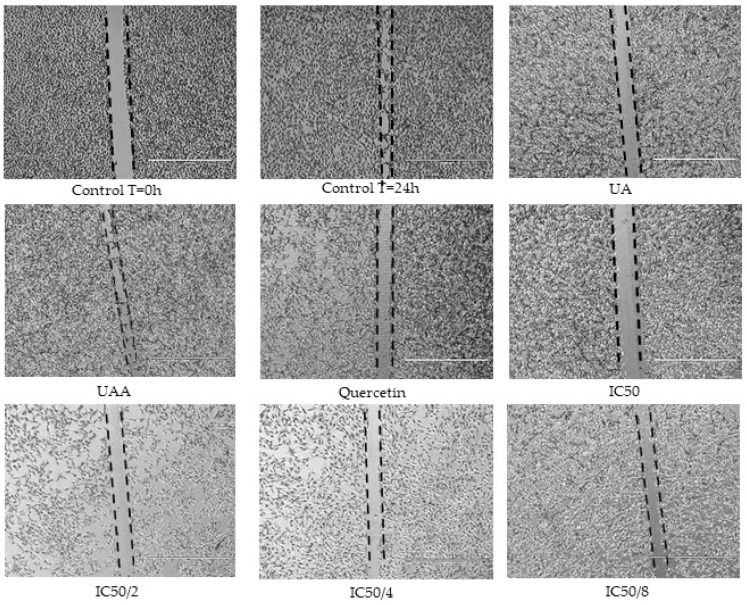
2D anti-migratory effects of 3-*O*-acetylursolic acid (UAA) and ursolic acid (UA) on B16-F10 murine melanoma cells alone and in fixed ratio combinations with quercetin. Representative microscopy images showing gaps in a confluent cell monolayer immediately after scratching (0 h) and at 24 h post-wounding (n = 3).

The anti-migratory properties were evaluated using scratch assay where a scratch was first created in a monolayer of B16-F10 melanoma cells and the cells were treated with the test drugs. The anti-migratory capacity of the single compounds was evaluated and their IC_50_ calculated (Table 1) as shown by gap closure in Figure 8. Notably, 3-*O*-acetylursolic acid did not inhibit melanoma migration, suggesting that the presence of the acetyl group diminishes the anti-migratory properties of the parent molecule. 3-*O*-acetylursolic acid was not active in this model at concentrations up to 200 µM (Data not shown). We therefore further tested the in vitro anti-invasive properties (3D migration) of ursolic acid and quercetin in a Matrigel-coated Transwell model. 

### 3.7. Effects of the Combination Treatments

Combination treatments of the triterpene acids and quercetin failed to show any synergy in suppressing cell proliferation (data not shown).

Due to the lack of anti-migratory activity of 3-*O*-acetylursolic acid, we only tested the combination of ursolic acid with quercetin in cells exposed to ‘fixed ratios’ as follows: 1, 1/2, 1/4, and 1/8 of their IC_50_s. Quantitative analyses of the relative migration rates are summarised in Table 1. The results show that the IC_50_/2, IC_50_/4, and IC_50_/8 combinations significantly decreased cell migration during the scratch assay (*p* < 0.05) and produced synergistic effects as indicated by the combination index (CI) value. 

**Figure 9:**
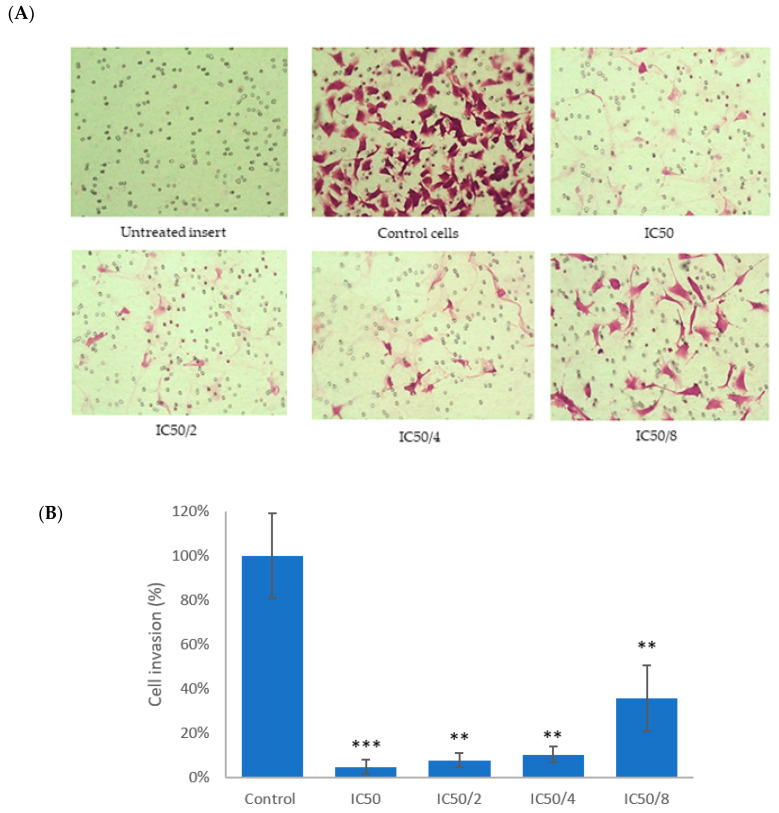
(**A**) Representative microscopy images (10×) showing invasive murine melanoma B16-F10 cells after treatment with quercetin and ursolic acid (48 h) using Transwell^©^ invasion assay. (**B**) Quantitative data of the 3D invasion experiment. Data are presented as the mean ± SD (n = 3), (**) *p* < 0.01; (***) *p* < 0.001.

The effect of the combinations at the same fixed ratios of both drugs on cell invasion is shown in Figure 9. All combination treatments down to 1/8 IC_50_s significantly suppressed 3D cell B16-F10 melanoma cells movement. The results indicated that we could reduce the concentrations of combined drugs to ¼ without any significant loss of anti-invasive effects when compared with full IC_50_s.

## 4. Discussion

The above-presented results evidence for the first time that the 3-acetylation of ursolic acid changes the phase at which A375 cells arrest their cell cycle. This occurs with a similar GI_50_ and sensitivity index to the parent molecule. The blockage at the S-phase results in dramatic morphological changes during the apoptotic phase not seen with ursolic acid, which arrests the cell cycle at the sub-G1 phase instead. 

Apart from this differential effect, the anti-proliferative effect of the two triterpenic acids follows a similar timeline: treatment with both compounds induced early signs of apoptosis within 24 h, as revealed by the elevated percentage of Annexin V-positive/ PI-negative cells (3.99% vs. 5.40%), respectively. This is followed 24 h later by increased caspase-3 activity and disruption in the balance between Bax and Bcl-2 expression in favour of Bax, thus suggesting the involvement of the mitochondrial pathway in apoptotic death. This is consistent with previous studies for ursolic acid on non-small cell lung cancer cells and M4Beu cells [7,27]. However, previous research has shown that changes in Bax protein are dependent on the cancer cell line [28,29]. 

Previous research on melanoma M4Beu cells had shown how ursolic acid induces cell arrest at sub-G1 or G1 phase (depending on the cell line) followed by mitochondrial-related apoptosis. Strikingly, the anti-proliferative effect at 24 h and 48 h is not matched by a reduction of mitochondrial viability [29,30]. A similar effect was described for the isoflavone genistein in MCF-7 cells [31]. The authors concluded that because genistein and UA are potent inhibitors of both tyrosine kinases and topoisomerase II, these targets were related to the observed discrepancy [7]. 

The S-phase arrest induced by 3-*O*-acetylursolic acid is still compatible with the inhibition of topoisomerase II, as seen by the effect of ciprofloxacin in COLO829 melanoma cells [32] suggesting that the 3-acetylation changes the expression of cyclins and CDKs involved in S phase progression. As DNA is replicated once during the S-phase, cells can neither go forward nor retreat to G1 stage [33]. This differential effect may be the cause of the dramatic changes induced by 3-*O*-acetylursolic acid in cell morphology compared to ursolic acid-treated cells, featuring significantly higher cell shrinkage and membrane blebbing.

Melanoma is highly metastatic cancer. Ursolic acid has been reported to inhibit melanoma migration and angiogenesis [9] but we found here that 3-*O*-acetylursolic acid is devoid of any significant effect on B16F10 cell migration, suggesting that the hydroxyl group is likely to be essential for the anti-migratory effect of ursolic acid on these cells. 

There is increased interest in combinatorial treatments of chemotherapy drugs with quercetin [20]. Previous work described how quercetin and sulforaphane were able to reduce melanoma growth in a mouse model by suppressing MMP-9 expression in the mouse tumours [34]. As ursolic acid and quercetin have been reported to display anti-migratory activity on melanoma cells, the potential effects of combining both compounds on melanoma migration and invasion have been investigated. The results revealed synergistic effects when UA combined with quercetin at a ratio 9:1. This combination markedly reduced melanoma invasion at all tested concentrations, thus allowing for a reduction of the required concentration up to four times less than their original IC_50_s. However, no anti-proliferative synergistic effects were found between the triterpene acids and quercetin. In a natural products discovery context, the ubiquity of these two compounds may explain the decrease of anti-migratory in vitro activities when many crude extracts are fractionated into increasing polarity fractions. 

## 5. Conclusions

In summary, 3-acetylation of ursolic acid maintains the potency and overall apoptotic mechanism of the parent molecule with a more aggressive influence on the morphology of A375 melanoma cells due to their arrest at S instead sub-G1 phase. We also demonstrate here that ursolic acid can act in synergy with quercetin to reduce cancer cell migration.

## Figures and Tables

**Table 1:** Calculation of the synergy between ursolic acid and quercetin in the 2D migration assay. Data are presented as the mean ± SD (triplicates of three separate passages).

Ratios	Quercetin µM	Ursolic Acid µM	Relative Migration	Combination Index	Relative Synergy ^1^
n/a	125	0	50%	n/a	n/a
n/a	0	13.7	50%	n/a	n/a
IC_50_	125	13.7	24%	0.90-1.10	±
IC_50_/2	65.5	6.85	34%	0.7-0.85	++
IC_50_/4	31.25	3.42	44%	0.3-0.7	+++
IC_50_/8	15.62	1.71	52%	0.3-0.7	+++

^1^ (±) Nearly additive; (++) Moderate Synergism; (+++) Synergism [26].
